# New Developed Cylindrical TM_010_ Mode EPR Cavity for X-Band In Vivo Tooth Dosimetry

**DOI:** 10.1371/journal.pone.0106587

**Published:** 2014-09-15

**Authors:** Guo Junwang, Yuan Qingquan, Cong Jianbo, Ma Lei, Dong Guofu, Yang Guoshan, Wu Ke

**Affiliations:** Beijing Institute of Radiation Medicine, Beijing, China; Martin-Luther-Universität Halle-Wittenberg, Germany

## Abstract

EPR tooth in vivo dosimetry is an attractive approach for initial triage after unexpected nuclear events. An X-band cylindrical TM010 mode resonant cavity was developed for in vivo tooth dosimetry and used in EPR applications for the first time. The cavity had a trapezoidal measuring aperture at the exact position of the cavity’s cylindrical wall where strong microwave magnetic field H1 concentrated and weak microwave electric field E1 distributed. Theoretical calculations and simulations were used to design and optimize the cavity parameters. The cavity features were evaluated by measuring DPPH sample, intact incisor samples embed in a gum model and the rhesus monkey teeth. The results showed that the cavity worked at designed frequency and had the ability to make EPR spectroscopy in relative high sensitivity. Sufficient modulation amplitude and microwave power could be applied into the aperture. Radiation induced EPR signal could be observed remarkably from 1 Gy irradiated intact incisor within only 30 seconds, which was among the best in scan time and detection limit. The in vivo spectroscopy was also realized by acquiring the radiation induced EPR signal from teeth of rhesus monkey whose teeth was irradiated by dose of 2 Gy. The results suggested that the cavity was sensitive to meet the demand to assess doses of significant level in short time. This cavity provided a very potential option for the development of X-band in vivo dosimetry.

## Introduction

Radiation disasters such as Three Mile Island incident, Chernobyl and Fukushima stricken nuclear reactors, vividly illustrate that populations potentially expose to unknown doses of ionizing radiation, especially terrorisms such as the release of dirty bomb probably cause great panic and disruption in public [1]. A possible radiation disaster may involve large number of victims who are potentially exposed to significant ionizing dose. The rescue of these situations urgently depends on rapid and accurate triages that find people whose exposure magnitude would cause acute radiation syndrome (ARS) [2]. So it’s indispensable for government to establish the capability to determine individuals’ exposure dose after radiation event quickly and in situ.

Radiation induces radical materials in hard tissue, especially the radiation induced signal in human teeth would maintain measurable and stable even after quite a long time [3], [4]. Therefore EPR tooth dosimetry is widely recognized as a particularly attractive approach for initial triage after an unexpected nuclear event [5]. EPR tooth measurement has been studied for years and represents a very useful dosimeter in retrospective dosimetry [6]–[12], but it was not feasible for vivo applications due to the invasive sample process and laboratory conditions before.

In order to meet the desirable triage demand in nuclear disaster rescue, scientists are developing EPR spectrometer especially for in vivo dosimetry, quickly and in situ, which will offer an ideal tool for accident rescue and greatly expand the EPR applications [13]. EPR in vivo dosimeter of L-band based on toroidal resonator [14]–[16] is under development for some years and has achieved reasonable progress [17]–[23]. Compared with the technique of in vivo dosimeter in L-band, X-band EPR, as another potential approach or supplement method, has some characters which worth considering: 1) Higher theoretical signal noise ratio (SNR) for much higher frequency; 2) Less sample requirement; 3) Sufficient spectra-dose research based on the commercial X-band ESR spectrometers. While it doesn’t make much progress after Ikeya’s previous work [24], [25], the key problem blocking the development of in vivo X-band EPR is the sensitivity of the resonant cavity.

After re-engineering a TE_111_ mode cylindrical cavity based on the previous work of Ikeya [26], we further developed a novel cylindrical TM_010_ mode cavity which was more suitable for in vivo tooth measurement. TM_010_ mode cylindrical cavity has been rarely used in common EPR practice. However, the microwave distribution of this cavity has the superiority to be developed into an outside-measurement cavity for special usage of in vivo tooth dosimetry. Compared with TE_111_ mode cavity, TM_010_ cavity costs much shorter scan time and could acquired radiation induced signal from irradiated tooth of lower dose. It would be a significant expansion of current EPR cavity resonators and applications.

This TM_010_ mode cavity was designed and calculated according to theoretic functions, then simulated by finite element software to verify and optimize the parameters. This paper introduces the design principle, microwave configuration and the theoretical calculation of the design process, as well as the feature test by measuring DPPH and 18 intact incisor samples. Radiation induced EPR signal could be observed remarkably from 1 Gy irradiated intact incisor within only 30 seconds, which is among the best in scan time and detection limit of in vivo dosimeters. As a supplement experiment for comprehensive verification, the in vivo rhesus monkey measurement experiments demonstrated how the X-band spectrometer worked and proved this scheme had the potential to be developed into feasible dosimeters.

## Methods

### 1. Cavity dimension design

The resonant mode, frequency and unloaded quality factor Q were related to cavity’s dimension, manufacture process and cavity wall materials. The following conditions were taken into account when designing the cavity’s configuration: 1) the resonant frequency was about 9.6 GHz to match microwave bridge; 2) the resonant mode was TM_010_; 3) higher quality factor Q; 4) as small as possible.

Let the ideal right circular cylindrical cavity radius be *r* and the height be *d*. First think about the resonant frequency was 9.6 GHz. The relation between *r*, *d* and frequency *f* of ideal cylindrical resonator was referred by
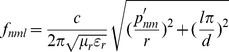
(1)


*ε_r_* was relative permittivity, *µ_r_* was relative permeability, *p_nm_^’^* was the m-th root of *J_n_^’^*, and *J_n_* was the *Bessel* function of the first kind. *n*, *m* and *l* were the numbers of half-wavelength variations in the standing-wave pattern of cylindrical TM*_nml_* mode [27].

TM_010_ mode, among all the modes in cylindrical cavity, worked at the lowest cutoff frequency when its dimension fitted certain conditions. This guaranteed the cavity works at designed lowest frequency, and the cavity was smallest in size for convenience of being placed in mouth. Let *n* = 0, *m* = 1, *l* = 0, *f* was a function of *r* dimension:
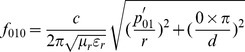
(2)


The lack of dependence of *d* about *f*_010_ meant that the resonant cavity could be made as narrow or wide (*d* dimension) accommodating to the magnet gap and teeth measurement. While taking into account that TE_111_ mode was usually regarded as the dominant mode in a cylindrical cavity, but TM_010_ mode worked at lower resonant frequency when:
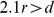
(3)


As a summary consideration: the *r* and *d* dimensions were selected to ensure its resonant frequency was 9.6 GHz. *d* dimension was slightly larger than the width of standard 3 cm wavelength waveguide (about 10.16 mm) for convenient manufacture and assemble. *ε_r_* and *µ_r_* were referred as air filling.

So, the equations were given by
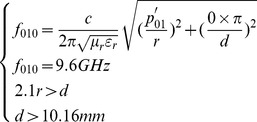
(4)


Function 3 showed that *f_010_* was only controlled by *r* but had no relation with *d,* therefore *d* was designed that the cavity worked at TM_010_ mode and was easy to manufacture and assemble as well as convenient to imbed into oral cavity. Let *d* be 12 mm, the solution of equations 4 were given by
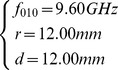
(5)


### 2. Calculations and Simulations

The cavity microwave distribution and resonant frequency was calculated by *Matlab* (Matlab is a software for technical computing). The constraint conditions were referred to 4. It turned out that the theoretical frequency was 9.58 GHz, quality factor Q under ideal conditions was 8000. And it indicated that 1∼3 mm in depth in the cavity was the most efficient depth of magnetic field H_1_.

The design was aided by finite elements simulation calculations. Its working condition was calculated by *HFSS* (HFSS is a microwave simulator by ANSYS corporation) using the following parameters: Material, silver; Filling dielectric, air; Mode, Eigen mode; Cavity Radius, 12 mm; Cavity Height, 12 mm; aperture size, 12 mm×3 mm.

The coupling unit was simulated by *HFSS* using the following parameters: Analysis, parameter sweep analysis; Sweep parameter, coupling size; Sweep range, linear step, from 1 mm×1 mm to 2 mm×8 mm, step was 0.1 mm×0.1 mm; Scan, frequency sweep from 9.5 GHz to 9.7 GHz, step was 0.01 GHz; Results, the scattering-matrix parameter S_11_ against frequency.

The aperture size was simulated by *HFSS* using the following parameters: Analysis, sweep analysis; Sweep parameter, aperture width; Sweep range, linear step from 1 mm to 8 mm, step = 1 mm; Results, eigen mode resonant frequency and quality factor Q.

### 3. Manufacture and Physical parameters

The cavity was manufactured by professional microwave instruments factory using copper material and polished rigorously and plated by silver for lower surface resistance. The wall’s thickness was about 1.5 mm so that it can supply enough structure strength. The resonant frequency and quality factor Q of the cavity were tested using reflection method by hp 8350B scan oscillator and hp 8757D scalar network analyzer.

### 4. In vivo EPR spectrometer

As the foundation of research, we designed an in vivo EPR dosimetry platform including clinical magnet which had relative large gap for in vivo use, modulation coils separated with cavity and fixed on magnet poles, alternating current power amplifier for modulation, signal receiver and data process unit and other relevant equipments. The homogeneity of the magnet was approximately 5×10^−4^ in a sphere area of diameter 10 mm. Microwave bridge was a Bruker-ER041 unit. Signal processor was the SR830 digital phase-locked amplifier of Stanford Research System. This platform offered in vivo detection conditions and made it possible to investigate the cavity performance.

### 5. Microwave power and modulation amplitude measurement

DPPH(1,1-Diphenyl-2-picrylhydrazyl radical 2,2-Diphenyl-1-(2,4,6-trinitrophenyl)hydrazyl) sample was used for signal intensity calibration. DPPH powder was inserted in quartz capillary to form a standard sample of 1 mm in length and 1 mm in diameter. The DPPH sample was fixed in the centre of the cavity’s aperture. Typical experiments conditions were: microwave power 1∼200 mW and fixed at 1 mW when modulation amplitude changed to obtain modulation feature, modulation amplitude 0.11 mT and fixed at 0.1 mT when microwave power changed to obtain microwave power feature, scan time 20 s, time constant 0.04 s, scan width 10 mT, centre field 0.34 T, scan magnetic field width 10 mT.

### 6. Tooth measurement

All measurements were performed on a homemade in vivo spectrometer platform. The samples were 18 pieces of irradiated human incisors which had no significant dental cavities or metal filling or history of endodontic treatment. All the teeth were collected from departments of stomatology in hospitals. They were removed as part of routine medicine care. These teeth were abandoned by patients and anonymized. The author had no contact with the patients. Teeth samples were separated into 6 groups, each group had 3 teeth of different volume. Incisors of different groups were irradiated by ^60^Co radiation source with different doses of 0 Gy, 1 Gy, 2 Gy, 4 Gy, 6 Gy, 8 Gy.

In order to simulate the in vivo measurements, the teeth were immersed in physiology saline. The teeth were wiped dry and fixed in gum model when measured. Only the cusps of the incisors were fixed into the cavity’s aperture, thus only the enamel part of the tooth was detected. The detection length of the teeth was controlled at about 2 mm, and the detected width and thickness of all teeth’s were recorded. Typical ERP measurement conditions were: scan time, 20∼300 s; scan width, 10 mT; microwave frequency, 9.5 GHz; microwave power, 20 mW; time constant, 0.03 s to 0.1 s.

### 7. Monkey in vivo measurement

The experiment animal was a rhesus monkey, 2 years old and conformed to the quality control standard of GB14922.1 and GB14922.2. The housing of the animals, design of the experiments, and other conditions, were approved by the animal welfare ethics committee, which conformed to the legal regulations of China in the responsible conduct of animal studies. The full name of the ethics committee was *BIRM animal welfare ethics committee*. The license No. was *IACUC of BIRM-2013011*.

The monkey was housed in conventional monkey house of animal centre. The conditions were: 24 hours negative pressure ventilation, room temperature 18∼24 centigrade, relative humidity 40%∼70%. The monkey house was twice water washed every day. The food was standard monkey feed offered by animal centre and the drink was disinfected cool water. Toys were applied to the monkey for environmental enrichment.

The monkey was anesthetized with 3% sodium pentobarbital (1 ml/kg). The monkey’s teeth were partially irradiated with dose of 2 Gy by ^60^Co radiation source. The monkey’s front teeth were cleaned up with distilled water, and then disinfected by 75% alcohol solution. The monkey and the microwave cavity were fixed at proper position and posture to make sure convenient measurement therefore the monkey could bit into the cavity aperture by its incisor cusp. The monkey was not sacrificed. The detection EPR parameters were: microwave power, 10 mW; scan time, 100 seconds; modulation amplitude, 0.2 mT; time constant, 0.03 s; center field, 338 mT; scan magnetic field width, 10 mT.

## Results

### 1. The cavity physics structure

**Figure 1:**
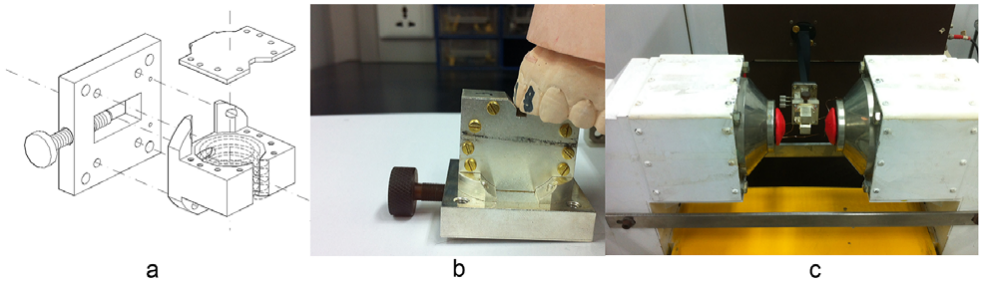
Structure of TM_010_ mode cavity resonantor and operation foundations. a: diagrammatic sketch of cavity resonantor. b: how the cavity aperture measured tooth. c: the cavity resonantor was assembled to a home-made in vivo spectrometer.

The cavity resonator was composed of cylindrical cavity, detection aperture, tune module and a lid assembled by screws. The tune module consisted of coupling hole and a dielectric screw with a metal tip to adjust coupling coefficient. There was a trapezoidal measuring aperture penetrating the metal wall at the exacted position where strong microwave magnetic field H_1_ concentrated and weak microwave electric field E_1_ distributed. Fig. 1a showed the resonant cavity diagrammatic sketch, and the dash line in the cavity demonstrated the microwave magnetic field inside the cavity. Microwave could leak into the aperture and apply to the teeth in the aperture. Trapezoidal profile allowed more volume of detected tooth without bringing about much microwave attenuation.

Fig. 1b showed the cavity after manufactured and assembled. The upper jaw model was used to demonstrate how the aperture measured incisors. Fig. 1c showed the cavity was connected to the microwave bridge of the in vivo spectrometer platform. The taper silvery magnet poles at cavity’s two sides were poles of the C-shape main electromagnet for applying static scan magnetic field (B_0_), and could comfortably and effectively encompass the human head chin. The distance between the magnet poles was 90 mm. The red coils attached to magnet poles provided modulated magnetic field B_m_, and was driven by specially designed alternating current amplifier.

### 2. The electromagnetic configuration

**Figure 2:**
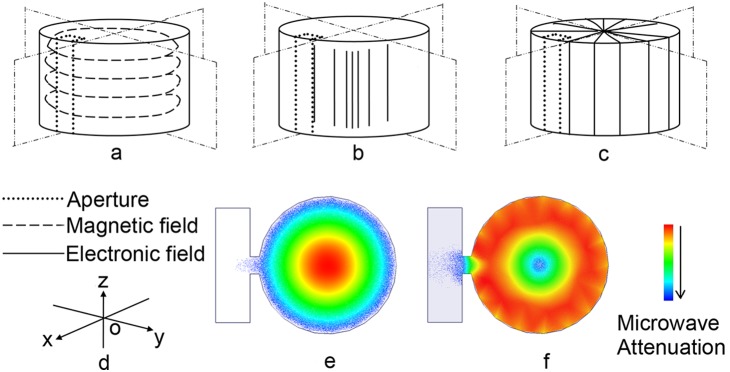
Sketches of electromagnetic field configuration and surface current distribution of cylindrical TM_010_ mode cavity. a:magnetic field H_1_ configuration. b:electric field E_1_ configuration. c:surface current flow distribution. d:coordinate system. The dashed rectangle in a, b and c refers to the measuring aperture position. e: Electric field E_1_ distribution in XY plane. f: Magnetic field H_1_ distribution in XY plane.

TM_010_ mode had been rarely used in regular EPR spectrometer for its electromagnetic distribution was not appropriate for conventional applications. However it applied an ideal option to make measurements when the sample was out of the cavity. Fig. 2 illustrated the sketches of electromagnetic field configuration and current flow distribution on the cavity inner surface. Fig. 2a showed that the magnet field H_1_ was paralleled to XY plane and most energy was distributed near the cavity cylindrical wall. Fig. 2b revealed the electric field E_1_ was paralleled to Z axis, and concentrated within the center area of the cavity. Fig. 2c showed the current flow distribution on the cavity’s inner surface wall. The electric field began and ended on induced charges on the plane face of the cavity, and currents in the walls perpendicular to the H_1_ direction was induced by the magnetic field H_1_ tangential to the cavity walls. The circuit flowed through the centers of the magnetic field loops, and induced charges on the XZ plane near the centers of these loops. The energy density of E_1_ reached the highest in the center of the cavity but was relatively weak near the cylindrical wall. Fig. 2e and Fig. 2f showed microwave distribution drawn by simulation software *HFSS* in the XY plane in middle cavity region. The simulation results suggested that: (1) the microwave electromagnet configuration was accordant with Fig. 2a and Fig. 2b; (2) the lowest mode in the cavity was TM_010_; (3) resonant frequency was 9.58 GHz, and (4) unload quality factor Q in ideal condition was about 6500. These results were quite close to our theoretical calculations of ideal resonant cavity. Besides, the next lower resonant frequency was 14.50 GHz, which leaved a sufficient safe region for single mode demand.

The dot-line rectangle in Fig. 2a, Fig. 2b and Fig. 2c indicated the position of measuring aperture. The geometry and position was designed by: (1) the microwave magnetic field H_1_ applied into the aperture was perpendicular to the scan magnetic field and modulation magnetic field; (2) the aperture was opened where there was strong H_1_ and weak E_1_ field; (3) the aperture’s longer edge was parallel to current flow on the inside cavity wall avoiding too much cutting of surface current flow. The first requirement arose from the nature of the resonance condition. The second requirement was because the amount of microwave energy absorbed by the sample was proportional to H_1_^2^ (before saturation) and the greater of the H_1_, the higher of the signal-to-noise ratio could acquire from spectra. And the third requirement minimized microwave energy loss [28].

### 3. Coupling unit and Detection aperture

**Figure 3:**
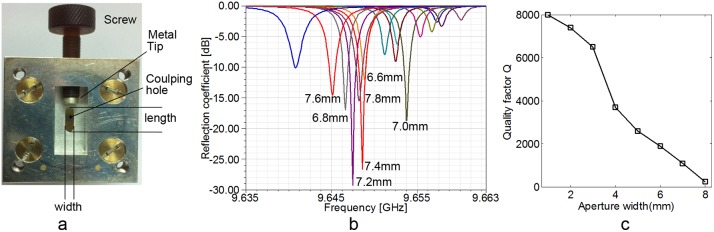
The design of the coupling unit, coupling hole and aperture geometry. a: the photo of coupling hole. b: The voltage reflection coefficient (scattering-matrix parameter S_11_) by computer simulation using coupling hole of different length. c: The resonant quality factor Q against different detection aperture width.

The resonant cavity was coupled to the waveguide by coupling hole. Fig. 3a showed the photo of the coupling unit, which was consist of coupling hole, adjusting screw with metal tip and a short waveguide. The coupling hole was a rectangular hole with round corner on the cavity cylindrical wall and was about 0.5 mm in thickness. The coupling constant was varied by adjusting a tunable dielectric screw with a metal tip immediately outside the hole. The metal tip was a cylinder whose radius was 2.4 mm and thickness was 1 mm. The distance between the center of metal tip and the coupling hole is 4.5 mm. The coupling hole was optimized by simulation software. Fig. 3b indicated the voltage reflection coefficient of the cavity with coupling hole of different size. The results showed the cavity could work at the best coupling status when the iris was 2 mm in width and about 7.2 mm in length.

The detection aperture’s geometry was inevitable for detection sensitivity. Fig. 1a and Fig. 1b showed the aperture profile. The cavity’s height and width was calculated and optimized by simulation software. Penetrating in Z dimension of aperture guaranteed the modulation magnet field applying into the aperture from outside coils. Wider aperture guaranteed more effective detection volume and space for tooth placement, while reduced the cavity resonant quality factor Q. Fig. 3c showed the theoretical resonant quality factor Q against aperture width. The Q reduced severely when the aperture width exceeded 3 mm. Finally, the aperture was trapezoid for more effective sample volume without resonant quality factor Q loss, and the trapezoid narrow edge was 3 mm at the inner cavity wall side while the wide edge was 8 mm at the side out of the cavity.

Simulation calculations could only roughly offer reference in designs, however the best coupling and aperture was determined by many factors. These parameters were optimized in the following real experiments under different parameters.

### 4. Cavity physical parameters

Some main physical parameters of the cavity were listed in Table 1. The cylindrical cavity was 12 mm in radius and 12 cm in height. Its average thickness was about 1.5 mm. The aperture size was 12 mm×3 mm (at the side of inner cylindrical cavity wall). Resonant frequency was around 9.5 GHz. The unloaded resonant quality factor Q was about 6500 while the loaded Q was about 3100. The size of coupling hole was 2 mm×7.2 mm. This cavity could reach good matching with EPR system in wide power range from 10µW to 200 mW by adjusting the coupling unit. These physics parameters indicated that the cavity and the coupling unit worked in designed and proper status.

**Table 1:** Some important parameters of TM_010_ mode cavity.

Items	TM_010_
Radius(mm)	12.00
Height(mm)	12.00
Aperture size(mm)	3 mm×12 mm
Loaded Q value	3100±100
Frequency(GHz)	9.50±0.05

### 5. The applying of microwave power and modulation amplitude in aperture

Experiments were carried out by measuring DPPH sample with different microwave power and modulation amplitude. Three characteristics of the cavity were interested: 1) Whether EPR signal could be acquired using this cavity and relevant equipments; 2) Whether sufficient microwave power could apply on sample in the aperture; 3) Whether sufficient modulation magnetic field could apply into the aperture.

**Figure 4:**
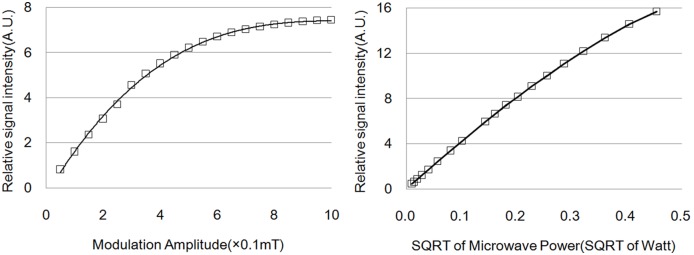
Relative signal intensity of DPPH sample against microwave power (square root or power) and modulation amplitude. The microwave power was the power generated by the microwave bridge. The modulation amplitude was that generated by the modulation coils in vacuum space.

As the result, the EPR spectrometer acquired strong signal of DPPH samples, certifying this homemade spectrometer worked well with this cavity. Fig. 4 illustrated respectively the relative signal intensity against square root of microwave power and modulation amplitude. The relative signal intensity increased as microwave power increased, and more microwave power contributed to signal intensity from 10 µW to 200 mW. The relative signal intensity increased as modulation amplitude increased before saturation, more modulation amplitude contributed to signal intensity from 0.05 mT to 0.5 mT. Higher modulation amplitude than 0.5 mT didn’t improve the signal intensity obviously but leaded to spectrum intensity saturation and deformation.

### 6. Teeth Samples Measurement

**Figure 5:**
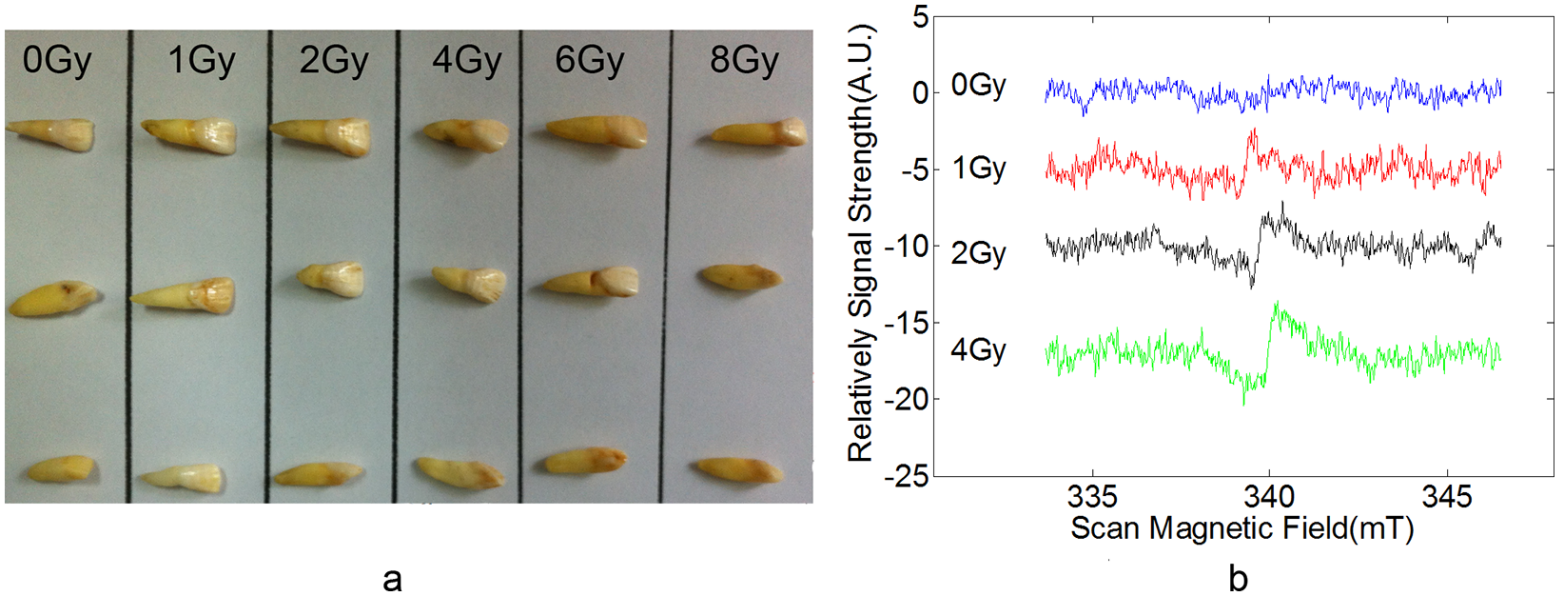
Tooth samples and spectra. a:Teeth samples. b: spectra from 0–8 Gy irradiated tooth within 30 s.

The measurements of intact incisors were performed to verify the applicability of in vivo tooth dosimetry using this cavity, and check its detection sensitivity and signal-dose response. The teeth were fixed in gum model to simulate in vivo measurement. The position and depth of the incisors into the aperture were controlled accurately, and then recorded their effective detection sample volumes. EPR spectra were recorded at routine laboratory environment. Fig. 5b showed spectra of 0–4 Gy irradiated teeth. The radiation induced signal from 1 Gy irradiated tooth could be acquired within only 30 seconds measurement. The results also showed that more radiation doses increased the signal intensity and improved signal–noise ratio, and longer scan time also resulted in signal intensity increasing and signal-noise ratio improving.

**Figure 6:**
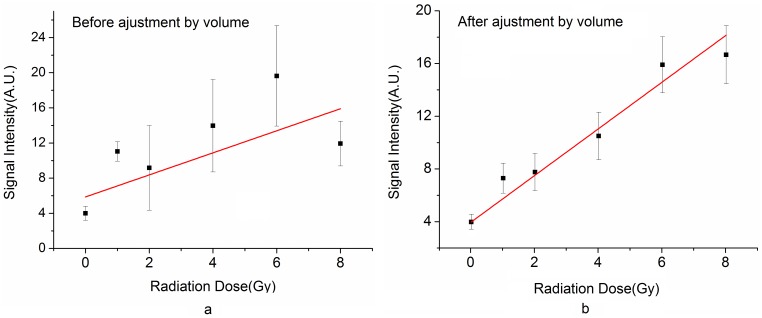
Dose response curve. a: Dose response curve without volume adjustment. b: Dose response curve after volume adjustment.

The in vivo EPR spectrometer with this cavity measured all the 18 irradiated teeth which were radiated with dose of 0–8 Gy (Fig. 5a showed the photo of all the teeth). The intensity of radiation induced signal were acquired and analyzed, and then this data formed the dose response curve. Considering the effective detection sample volume in the aperture would bring certain influence to the signal intensity, the dose response curve was optimized by rectifying the signal intensity according to sample volume. Fig. 6 demonstrated respectively the dose response curve before (Fig. 6a) and after volume adjustment (Fig. 6b). The linear correlation coefficient of dose response curve improved from 0.5270 to 0.9798 after volume adjustment.

### 7. In vivo measurement of monkey

**Figure 7:**
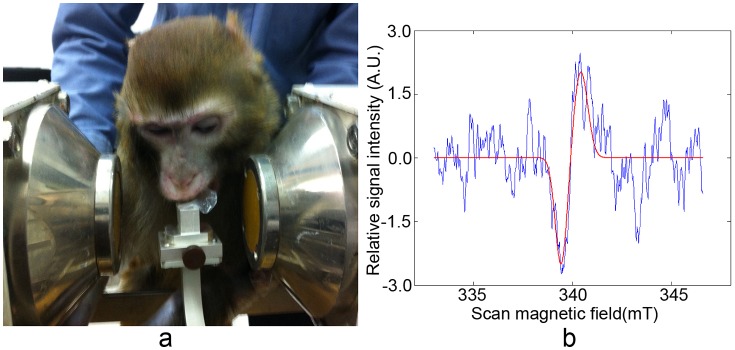
Application for in vivo monkey measurement (a) and acquired spectrum (b). The measurement parameter were: radiation dose, 2 Gy; Magnet gap, 90 mm; microwave power, 10 mW; scan time, 100 seconds; modulation amplitude, 0.2 mT; time constant, 0.03 s; center field, 338 mT; scan magnetic field width, 10 mT.

The rhesus monkey teeth were measured to verify its applicability of tooth dosimetry under the conditions that were most analogous to in vivo usage. The monkey was irradiated with dose of 2 Gy. Fig. 7a demonstrated that the monkey was fixed in the position that its upper incisors could bite into the aperture, and situated in the gap of magnet poles. Fig. 7b showed the EPR spectrum acquired from monkey teeth within 100 seconds scan. Hydroxyapatite was the main component of the tooth cusp. According to the g-value of hydroxyapatite, the center position of the main radiation induced signal was at 0.3391±0.0001 T. The fitting curve in figure 7b was fitted at the proper position. Radiation induced signal was observed from the spectrum and there was relatively strong radiation induced signal intensity. This result indicated our cavity and the in vivo EPR dosimeter were potentially available for in vivo dosimetry.

## Discussion

This TM_010_ mode cavity was designed as the detection probe for in vivo tooth dosimetry. Apparently the most important future was whether it could perform in vivo measurement and sensitive enough to detection weak radiation induced signal in irradiated tooth. X-band in vivo dosimetry was usually regarded infeasible for its microwave attenuation caused by water and lower cavity sensitivity since Ikeya’s effort. In order to confirm that, we measured teeth fixed in a gum model, and were immersed in physiological saline before measurement to simulate the moist environment in human oral. Most notably, the detection area in our method is the incisor cusp, which is mostly made of enamel instead of dentine and contains very little water and brings little microwave attenuation. The soft tissue such as the dental bed is far from the detection aperture, the dielectric loss from which could be ignored.

The most fascinate advantage of X-band in vivo dosimetry is the higher detection sensitivity. Its radiation induced signal is much easier to distinguish and analysis. L-band EPR dosimeter, such as the apparatus developed in Dart-Dose CMCR, can detect tooth spectra in less than 300 seconds data acquisition to estimate dose with standard error of less than 1 Gy [19]–[21]. Recently C-band pulsed in vivo spectrometer detects teeth of 4 Gy dose irradiated within 114 seconds of signal acquisitions, and its sensitivity is insufficient for practical applications [29]. According to our results, X-band may achieve higher sensitivity within less than 30 seconds, which is an obvious improvement of the triage efficiency in emergency scene.

How scan magnetic field and modulation magnetic field applies is another obstacle for X-band in vivo condition. We improved and redesigned the scheme of X-band in vivo spectrometer, including the cavity, C-shape magnet, modulation amplitude and coils, signal process unit and so on. The modulation magnetic field was excited by coils far away from the cavity, which helped to simplify the cavity structure and improved resonant quality factor Q. During the experiments all the spectra were acquired by this spectrometer and indicated this scheme was quite feasible. We measured a rhesus monkey tooth to initially but comprehensively check our scheme and the feasibility of in vivo spectrometer. Systematic in vivo measurement experiments, such as dosimetry of patients who received radiotherapy or volunteers whose tooth is replaced by an ionized tooth of known dose, will be carried out in the future investigation.

The signal-dose relation is a rather complicated procedure. The signal intensity is influenced by many factors such as radiation dose, tooth age, tooth size, tooth angles, detection position, volume alignment, spectrometer parameter and so on. We measured the tooth size and rigorously controlled the effective detection volume to insure the signal intensities of different teeth comparable. More precise detection experiment will be carried out in the future by adding reference sample in the cavity and by some other implements.

TM_010_ mode cavity has not been reported in EPR study before, and this novel approach also expands EPR resonant cavity family. Our initial work mainly focuses on the development of more sensitive cavity. Further detailed design of cavity will focus on aperture geometry considering incisors match, structure simplify, cavity filling factor, and reference signal label. The future research of spectrometer will also pay more attention on spectrometer improvement, human in vivo measurement experiment and signal-dose effect.
